# Application of Deep Learning Networks to Segmentation of Surface of Railway Tracks

**DOI:** 10.3390/s21124065

**Published:** 2021-06-12

**Authors:** Piotr Bojarczak, Waldemar Nowakowski

**Affiliations:** Faculty of Transport, Electrical Engineering and Computer Science, University of Technology and Humanities, Malczewskiego 29, 26-600 Radom, Poland; w.nowakowski@uthrad.pl

**Keywords:** deep neural networks, image segmentation, detection of elements of railway track

## Abstract

The article presents a vision system for detecting elements of railway track. Four types of fasteners, wooden and concrete sleepers, rails, and turnouts can be recognized by our system. In addition, it is possible to determine the degree of sleeper ballast coverage. Our system is also able to work when the track is moderately covered by snow. We used a Fully Convolutional Neural Network with 8 times upsampling (FCN-8) to detect railway track elements. In order to speed up training and improve performance of the model, a pre-trained deep convolutional neural network developed by Oxford’s Visual Geometry Group (VGG16) was used as a framework for our system. We also verified the invariance of our system to changes in brightness. To do this, we artificially varied the brightness of images. We performed two types of tests. In the first test, we changed the brightness by a constant value for the whole analyzed image. In the second test, we changed the brightness according to a predefined distribution corresponding to Gaussian function.

## 1. Introduction

A steady increase both in tonnage and people carried by trains requires that an emphasis be put on the state of the railway track infrastructure. Track maintenance is a crucial task that has an immense impact on the safety of railway traffic. Many train derailments are caused by the poor shape of track elements, such as sleepers and elements fastening the rail to the sleepers. Every accident can generate heavy financial losses. Therefore, continuous monitoring of track infrastructure is very important from the point of view of safety. The condition of railway track components also has a significant impact on passenger comfort [1]. In addition, the poor condition of the track deteriorates the vehicle-infrastructure interaction performance [2,3]. Damage to the rails is caused by the load transferred, the weather conditions, and the dynamic interactions of the wheel and the rail. The main damages are rail corrugation, head checking and squats [4]. Surface diagnostics of individual elements, including the rails, makes it possible to determine their technical condition. For this purpose, different methods are used, one of them being the vision method [5,6,7]. Recent advances in CMOS imaging technology and Graphics Processing Units have resulted in commercial line-scan cameras and highly efficient graphics servers that can be used as elements of a visual system to inspect railway track elements. Such a system can be installed, for example, on an ultrasonic flaw-detection carriage or a track geometry car and constitutes the framework for implementation of advanced visual algorithms. The paper presents a method which allows for the detection of elements of the track. The authors use a Fully Convolutional Network with 8 times upsampling (FCN-8) to segment the image of railway track into regions containing ballast, sleepers, fasteners, rail, and turn-out. The system is able to distinguish between wooden and concrete sleepers. It can also detect four types of fasteners. In addition, the effectiveness of the proposed solution has been tested for different levels of brightness of railway track images. Our contributions are:

- application of FCN-8 network to detection of elements of track; the authors did not find in the available literature any information about the use of FCN-8 to detect elements of the railway track;
- evaluation of FCN-8 network performance at different levels of light intensity of railway track images; and
- evaluation of FCN-8 network performance at different distributions of light intensity of railway track images.

## 2. Related Works

Over the last two decades, visual systems have become readily present on the railway market. At first, these systems were only able to record images of the track. These images were reviewed by trained staff. Next, more advanced systems appeared. Many papers concerning algorithms for detection of railway track elements can be found in the literature. Reference [8] presents an algorithm for fastener detection in the track. It uses filter banks, which are directly convolved with the image under analysis. Each type of fastener is detected by a dedicated filter in which the coefficients are calculated using an illumination-normalized version of the Optimal Trade-off Maximum Average Correlation Height filter. According to Reference [8], the proposed algorithm allowed detecting the fasteners with an accuracy of over 90%. In Reference [9], the authors present methods which recognize such elements of track as sleepers, tie plates, spikes, and anchors. Sleepers and tie plates are detected using an edge filter along with Hough transform. Spikes and anchors are recognized using AdaBoost algorithm. Reference [10] uses Haar wavelets as features describing fasteners in the image of track. They are detected based on features by a classifier (detector) built using a probabilistic graphical model (PGM). Reference [11] uses Histogram of Oriented Gradients (HOG) as features and Support Vector Machine (SVM) as a classifier to detect fasteners in the image. References [12,13] allow detecting rails, sleepers, and tie plates. It uses Canny filter, Hough transform, and morphological operations to extract the features and a decision tree (C4.5 algorithm) as a classifier. Yet another work, Reference [14], uses distribution of the average energy of vertical detail coefficients for 4th decomposition level of 2D wavelet transform of the image to localize the rail in the image. Reference [15] uses Gabor filter bank to extract features and SVM as a classifier to segment wood sleepers from ballast. In these papers, the features are manually extracted (selected). In addition, the detector is built in two separate stages: feature extraction and classifier construction. This process is called shallow learning. The main problem in such an approach is the limited set from which features are extracted, which, in turn, can sometimes lead to feature selection which imprecisely describes the detected objects. The appearance of the paper by Reference [16] concerning the application of a deep convolutional network to object detection was a breakthrough in the field of machine learning. A deep neural network creates a set of features of the size much larger than for shallow learning, practically limited by the size of the image under analysis. In addition, feature extraction and classifier construction are performed in one stage during the learning process and features are selected independently of the user. Since then, many researchers have tried to apply deep neural networks to visual inspection of the railway track. Reference [17] applies a fully convolutional network which composes of 4 convolutional layers to grayscale images of railway track. It allows the detection of 10 types of textures including ballast, rail, wooden and concrete sleepers. Reference [18] also uses fully convolutional network consisting of 4 convolutional layers to segment the image. Additionally, it recognizes fasteners and classifies them into classes containing normal and broken fasteners. The classification is carried out by a combination of a convolutional network with SVM. In Reference [19], pre-trained faster R-CNN segments track surfaces and clamps. This segmentation is further used to identify anomalies. Next, Generative Adversarial Network (GAN) was applied to cluster normal and anomalous observations. Another paper, Reference [20], presents an application of deep networks (AlexNet and ResNet) to recognition of fasteners defects.

In the last few years, many papers concerning deep neural networks have been published [21,22,23,24,25,26,27]. Most of them concern the development of deep neural architectures for detecting objects in images; however, we will limit our discussion to the state of art architectures. Reference [28] presents a You Only Look Once (YOLO) network. The network sees the entire image during training and test time, which, in turn, allows for encoding of contextual information about detecting objects and their appearance. The image under analysis is divided into cells. Each cell proposes potential bounding boxes. The match between a box and detected object is determined by a confidence score in which the value is calculated using features generated by a convolutional network. With a single image processing over a network, it is possible to significantly reduce the processing time of the algorithm, reaching up to 50 frames per second (fps). However, the limited number of boxes proposed by the algorithm for the analyzed image reduces detection accuracy. Another fast network—Single Shot Multibox Detector (SSD)—is presented in Reference [29]. Unlike YOLO, SSD network adds several feature layers to the end of a base network. It allows predicting offsets to default boxes of different scales and aspect ratios and their associated confidence scores. This change increases the accuracy of the detection, as well as the speed of operation, to 58fps. Another network [30] called Faster R-CNN is composed of two sub-nets: Region Proposal Network (RPN) and a second network which performs the final detection of an object in the area indicated by RPN. Despite its high detection accuracy, it processes images at a relatively low rate of 5 fps. In all the above networks, the position of the detected object is marked by a bounding box centered on this object. A Mask R-CNN network [31], apart from generating a bounding box, also creates a mask for the detected object. The whole detecting process is split into two stages. First, the network with an architecture very similar to Faster R-CNN detects the object and marks it with a bounding box. Next, another network performs a semantic segmentation in the area confined to the bounding box. As a result, each point in the bounding box is assigned an object label when it belongs to it or a background label when it does not belong to it. Mask R-CNN operates at the rate of up to several frames per second. Another group of networks are those used only for semantic segmentation of images. It includes DeepLabv1 [32], DeepLabv2 [33], DeepLabv3 [34], DeepLabv3plus [35], and Fully Convolutional Network (FCN) [36]. The architectures of DeepLabv1 and DeepLabv2 are similar, both of them use a Deep Convolutional Network along with Atrous Convolution and Fully Connected Conditional Random Field (CRF) to segment the image. Additionally, DeepLabv2 is equipped with Atrous Spatial Pyramid Pooling (ASPP) module, which allows for increasing the accuracy of segmentation. ASPP integrates features from multiple scales so that objects of different sizes can be easily identified. DeepLabv3 is a modified version of DeepLabv2, in which cascaded modules with Atrous convolution are replaced with a single module executing ASPP. In DeepLabv3, a CRF module has also been removed. In order to further increase accuracy, in the newest version, called DeepLabv3plus, the decoder module has been added. It is achieved by concatenation of an upsampled version of a feature map coming from the ASPP module with an upsampled low-level feature map coming from the deep convolutional network. Despite its relatively high accuracy, they process the image at the rate of 8 FPS, which makes them unable to operate in real time.

## 3. Proposed Approach

In the proposed approach, it is possible to detect the elements of the track and determine the degree of the sleeper coverage by the ballast. The quality of the segmentation process is closely related to the complexity of the network structure, which, in turn, affects the speed of segmentation. Therefore, the choice of network is a tradeoff between the quality and speed of the algorithm. DeepLab networks are characterized by high accuracy at the expense of speed (up to several frames per second). On the other hand, FCN (Fully Convolutional Network) networks are characterized by lower segmentation efficiency, but provide higher speed of operation. There are three types of networks: FCN-32, FcN-16, and FCN-8. Numbers 32, 16, or 8 are called output stride. For example, output stride = 32 means that the spatial resolution of the final feature maps is 32 times smaller than the input image resolution. Larger size of feature maps corresponds to higher accuracy of segmentation process, which, in turn, has an impact on larger network structure and lower speed of operation. In order to select the networks, the result of their performance (based on the available track images) was compared; see Table 1. Pixel accuracy is calculated as a mean of all track elements.

**Figure 1:**
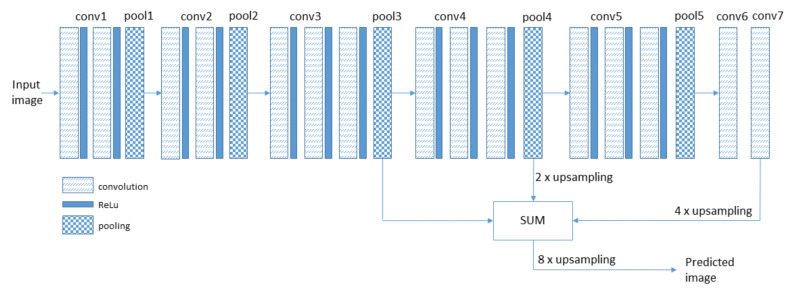
Block diagram of FCN-8.

We decided to use the FCN-8 network [36]. It performs semantic segmentation of the image. During this process, the network assigns the label to every pixel in the image. Its value depends on the object to which the pixel belongs. The main framework for this network is Convolutional Neural Network (CNN). There are three main architectures of CNN: AlexNet, VGGNet, and GoogLeNet. According to Reference [36], VGGnet has the highest segmentation accuracy among the aforementioned networks; therefore, it has been selected for our system. VGGNet, also called VGG-16, consists of the stack of convolutional, ReLu, and pooling layers followed by three fully connected layers. The convolutional layer performs an operation defined as:

(1) $$B\left(i,j,z\right)=\left(\sum_{k=\frac{-(K-1)}{2}}^{\frac{K-1}{2}}\sum_{m=\frac{-(K-1)}{2}}^{\frac{K-1}{2}}\sum_{n=0}^{N}A\left(i+k,j+m,n\right)*W\left(k+\frac{K-1}{2},n+\frac{K-1}{2},n,z\right)\right)+b\left(z\right),$$

where: *i* ∈ (0,...,P−1), *j*∈ (0, R−1), *z*∈ (0,...,Z−1) are the coordinates of the output matrix ***B*** of the layer along the X, Y, Z axes, respectively, ***A*** is the input matrix of the layer of the size *P × R × N*, and ***W*** is the weight matrix of the size *K × K × N × Z*. *K* determines the filter/kernel width in which the value is odd, *Z* corresponds to the number of filter used in this layer, and *b* is a bias vector of the size *Z*. The submatrix of the matrix ***W*** of the size *K × K × N* corresponds to the single neuron which is connected to the limited number of inputs determined by *K × K × N*. This layer performs the operation ***B*** = f(***A***, ***W***) which transforms the matrix ***A*** of the size *P × R × N* into the matrix ***B*** of the size *P × R × Z*. In case of the first layer of the network, the matrix ***A*** corresponds to the input image and N = 3 (R, G, B components of the image). The output matrix ***B*** is given to nonlinear activation function ReLu in the form of ***C*** = max (0, ***B***). Additionally, every few convolutional layers ***C*** matrix of the size *P × R × Z* is reduced to *P/2 × R/2 × Z* by subsampling or pooling [36]. The last three layers are fully connected layers. It means that every neuron of this layer is connected to all inputs of the previous layer. The last layer consists of 1000 neurons. Each neuron in this layer is responsible for the indication of one class of objects occurring in the image under analysis. According to Reference [36], a fully connected layer can be viewed as a convolutional layer with filter/kernel which cover the entire input regions. As a result, a coarse map of the size H/32 × G/32 × 1000 is created, where H and G denote the height and the width of the analyzed image, respectively. A simple straightforward method performs a 32 × upsampling of the coarse map to the size of the original image. However, the obtained result is also very coarse. In order to improve segmentation accuracy, information coming from a few earlier layers is added to the coarse form. According to Reference [37], upsampling operation can be performed using transposed convolution. FCN-8 sums the 2 × upsampled conv7 layer with pool4 layer. Next, the result is upsampled 2 times and sums with pool3 layer. The final segmentation map is created by 8 × upsampling of obtained result; see Figure 1.

All parameters (weights) of the network are learned during supervised training. While training the loss function, *L*, defined as a cross-entropy, is minimized:

(2) $$L=-\frac{1}{S}\sum_{i=1}^{S}P\left(x_{i}\right)logQ\left(x_{i}\right),$$

where: $P(x_{i})$ is the probability that determines to which class pixel $x_{i}$ belongs. This value is calculated on the basis of the training data. $Q(x_{i})$ is also the probability that determines to which class pixel $x_{i}$ belongs; however, its value is generated by the FCN-8 network during learning. *S* is the total number of training data given to the network during the learning process. When the loss function is near 0, then both probability distributions are almost the same (p(x)≈q(x)), and the learning process is stopped.

## 4. Experimental Results

We decided to use transfer learning, which means a model developed for a task is reused as the starting point for a model on a second task [38]. Such approach can significantly speed up training and improve the performance of our model (network). We used a pre-trained VGG-16 network as a framework to develop our visual system based on FCN-8. The network has been built using Tensorflow package.

### Dataset

**Figure 2:**
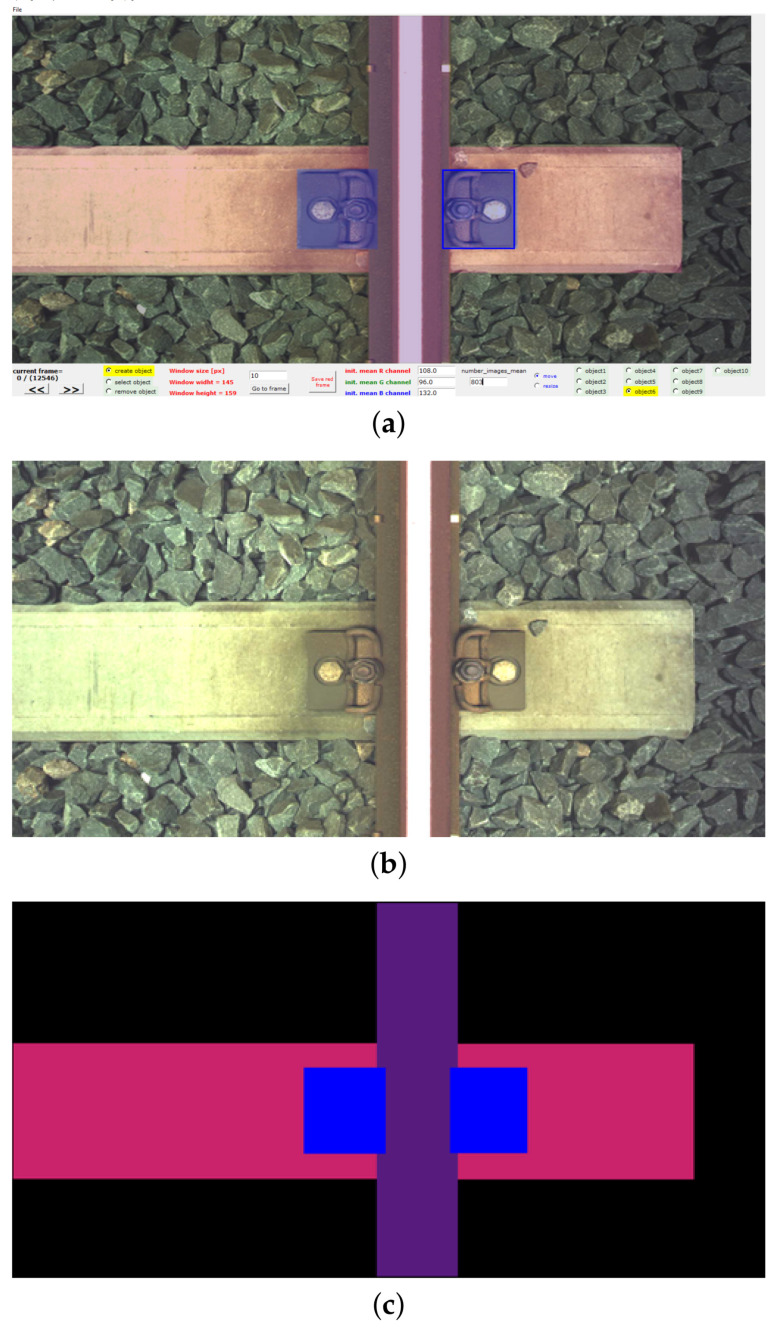
Print-screen of the tool and an example of the pair of images generated by the tool: (**a**) print-screen of the tool, (**b**) the image of the track, (**c**) the image with the ground truth labeled mask corresponding to the image in (**b**).

The images of the railway track were recorded in the form of video files. We used two line-scan cameras, which were mounted under the floor of the diagnostic carriage. Cameras are located perpendicular to the track. One camera covers the left half of the track and the other camera covers the right half of the track. Cameras were mounted on regular inspection carriage used to measure track geometry. Each of the cameras is connected to an associated video server via a dedicated 1GB Ethernet link. The system makes it possible to record video frames at carriage speed of up to 60 km/h. Due to the large number and size of video files, we have implemented customized software tools which allow the user to efficiently visualize and annotate the images. This tool has been implemented in Python using OpenCV library. Figure 2 presents the print-screen of the main page of this tool and an example of the pair of images generated by this tool.

The network is able to detect the following elements of the track: four types of fasteners, wooden and concrete sleepers, rails, and turnouts. The fastener group includes the following types: SKL rail fastening system, hereinafter referred to as *fastener1*; KS rail fastening system, hereinafter referred to as *fastener2*; WJ-7 rail fastening system, hereinafter referred to as *fastener3*; and KPO rail fastening system, hereinafter referred to as *fastener4*. To speed up the detection process, each camera is assigned one FCN-8 network. Because every network should work regardless of which camera it is connected to, therefore, the network is trained on the images recorded by both cameras. In addition, the training data has been augmented by randomly vertical mirroring of the recorded images. We collected: 5000 examples of *fastener1*, 5000 examples of *fastener2*, 5000 examples of *fastener3*, 5000 examples of *fastener4*, 5000 examples of wooden sleepers, 5000 examples of concrete sleepers, 7000 examples of rails, and 3000 examples of turnouts. Examples belonging to each group have been randomly divided in the proportion 60%, 20%, 20% into three groups: training data, hereinafter referred to as *train_data*; validation data, hereinafter referred to as *valid_data*; and testing data, hereinafter referred to as *test_data*. The prepared data contains images recorded in different seasons of the year and in different weather conditions. We normalized the input data set so that its mean is at zero. This significantly improves convergence of gradient optimization. Therefore, the mean values for each channel (R, G, B) are subtracted from corresponding channel of every input image. These values are calculated based on all available input images. The size of the original image (the half of the track) is equal to 2048 × 966. In order to further speed up the detection process, we decided to resize the images to the size of 512 × 256.

## 5. Performance of the Network

**Figure 3:**
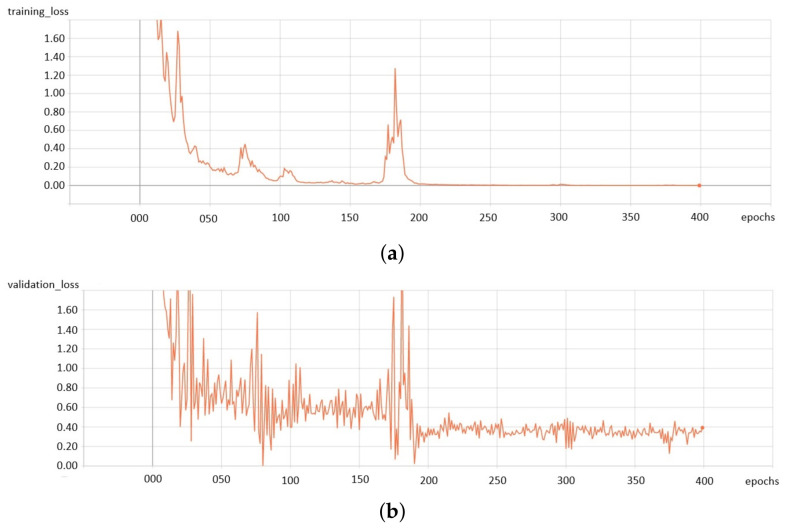
Loss error during the learning process: (**a**) for the *train_data* set, (**b**) for the *valid_data* set.

The network has been trained for 400 epochs on NVIDIA GeForce GTX 1080 Ti GPU. We used AdamOptimizer with a learning rate of 0.0001 to minimize the loss function. While training, two groups of data have been used: *train_data* for training and *valid_data* for testing. Figure 3 shows the loss error during the learning process for the *train_data* set (Figure 3a) and the *valid_data* set (Figure 3b).

After training, the performance of the network was verified on a *test_data* set. We used four pixel accuracy calculates metrics for this, namely: pixel accuracy, intersection over union, precision and recall. Pixel accuracy calculates percent of pixels in the image which were correctly classified. It is commonly reported for each class separately. When considering the per-class pixel accuracy, we are essentially evaluating a truth mask and this metric can defined as:

(3) $$Pixel\_accuracy=\frac{TP+TN}{TP+TN+FP+FN},$$

where: *TP* true positive represents pixels that are correctly predicted to belong to the given class (according to the target (truth) mask), *TN* true negative represents pixels that are correctly identified as not belonging to the given class, *FP* false positive represents pixels that are predicted as belonging to the given class though they do not belong to this class, and *FN* false negative denotes pixels that are identified as not belonging to the given class though they belong to this class. The second metric, called Intersection over Union (IoU) and also referred to as the Jaccard index, is essentially a method to quantify the percent overlap between the target (truth) mask and our prediction output. IoU determines the number of pixels common between the target and prediction masks divided by the total number of pixels present across both masks:

(4) $$IoU=\frac{T\_M⋂P\_P}{T\_M⋃P\_P}=\frac{TP}{TP+FP+FN},$$

where: *T_M* denotes pixels belonging to the target mask, and *P_P* represents pixels that are predicted as belonging to the target mask. On the other hand, precision is calculated as the ratio of correctly classified pixels belonging to a class to the total number of pixels classified as belonging to that class:

(5) $$Precision=\frac{TP}{TP+FP}.$$

The last metric—recall—is calculated as the ratio of correctly classified pixels belonging to a class to the total number of pixels belonging to that class:

(6) $$Recall=\frac{TP}{TP+FN}.$$

Table 2 presents mean values of Pixel accuracy, IoU, Precision, and Recall metrics calculated for track elements.

**Figure 4:**
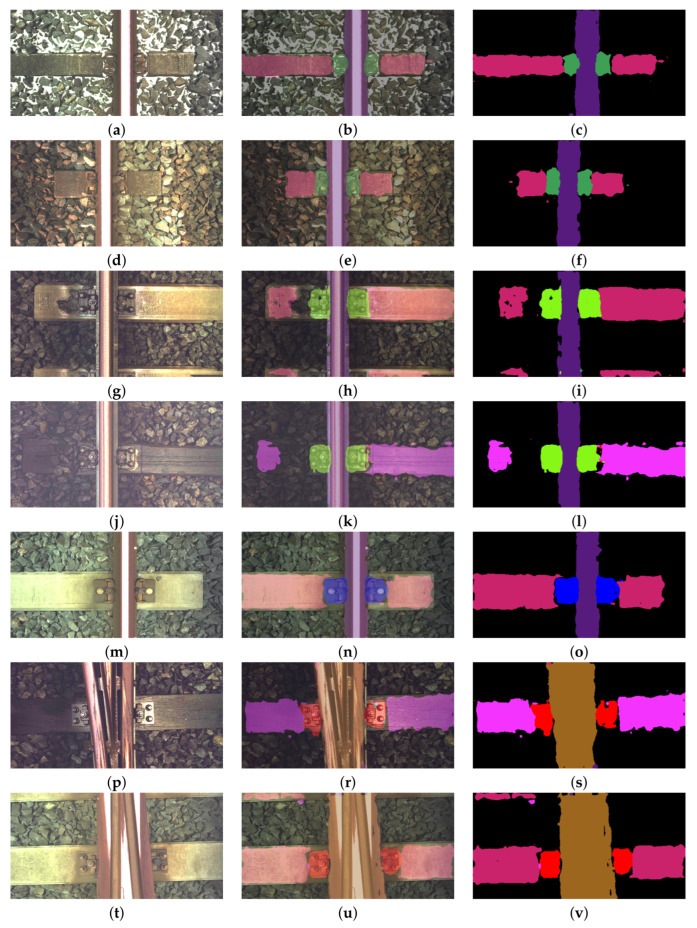
Examples of detection of track elements: (**a–c**), (**d–f**) *fastener1* and a concrete sleeper, (**g–i**) *fastener2* and a concrete sleeper, (**j–l**) *fastener2* and a wooden sleeper, (**m–o**) *fastener3* and a concrete sleeper, (**p–s**) *fastener4*, a wooden sleeper, and a turnout, (**t–v**) *fastener4*, a concrete sleeper, and a turnout.

The system presented in Reference [12] uses Canny edge extraction and Hough transform methods to extract feature. The types of the components are determined by using classification algorithm with decision trees. The average accuracy for this system was 0.83, while the average processing time was 166 ms. Reference [13] presents a system for turnout detection. In addition, this work uses Canny edge extraction and Hough transform methods to extract feature. The average accuracy for this system was 0.88, while the average processing time was 221 ms. Comparing the results, our algorithm is faster (67 ms) than those presented in the aforementioned works. In addition, the average accuracy of our algorithm was 0.891, which was higher than the work of Reference [12]. In addition, the accuracy of turnout detection was higher than in Reference [13]. Figure 4 presents examples of image segmentation for different elements of the track. The first column shows the image of the track, the second the image with the predicted mask overlaid on it and the last the predicted mask. The proposed FCN-8 network is able to segment the image even when the track is partially covered in snow; see Figure 4a–c. In addition, the network can also detect the situation when the sleeper is partially covered in ballast; see Figure 4d–l. The system presented here processes the single image at an average rate of 13 fps (GeForce GTX 1080 Ti GPU).

## 6. Invariance to Image Brightness

We also verified the invariance of the performance of the network to changes in brightness of the image. For this purpose, we artificially changed the brightness of each image belonging to a *test_data* set. We achieved this through conversion of RGB image model to HSV (Huge, Saturation, Value) model, in which the V component is responsible for the brightness of the image. We change the brightness of each image as:

(7) $$I=S*I_{nom},$$

where: *S* is a multiplier in the range of [0.1, 0.2, ..., 0.9, 1.0], and $I_{nom}$ is the brightness of each pixel in the original (unmodified) image. Each modified *test_data* set containing the images with predefined brightness level was given to the trained network. Next, an average pixel accuracy and IoU metrics were calculated for the images predicted by the network and related to the average pixel accuracy and IoU metrics obtained for the brightness level of $I_{nom}$(S = 1).

**Figure 5:**
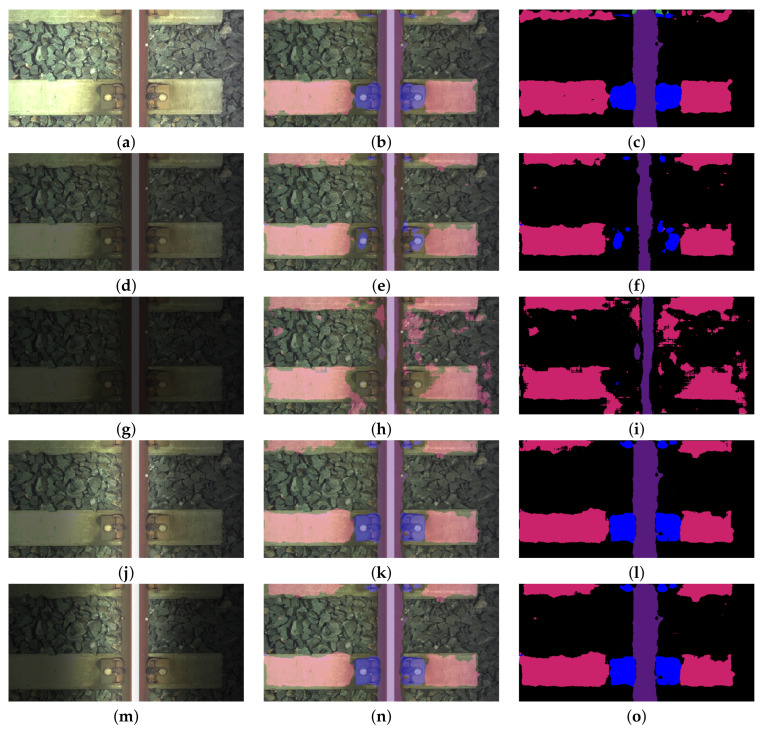
Examples of segmentation for selected images including concrete sleepers and *fastener3* elements: (**a**) the image of the track with S = 1, (**b**) the image with the predicted mask (S = 1) overlaid on the original image, (**c**) the predicted mask (S = 1), (**d**) the image of the track with S = 0.4, (**e**) the image with the predicted mask (S = 0.4) overlaid on the original image, (**f**) the predicted mask (S = 0.4), (**g**) the image of the track with S = 0.2, (**h**) the image with the predicted mask (S = 0.2) overlaid on the original image, (**i**) the predicted mask (S = 0.2), (**j**) the image of the track with the lowest multiplier in a mask vector = 0.4, (**k**) the image with the predicted mask (a multiplier in a mask vector = 0.4) overlaid on the original image, (**l**) the predicted mask (a multiplier in a mask vector = 0.4), (**m**) the image of the track with the lowest multiplier in a mask vector = 0.2, (**n**) the image with the predicted mask (a multiplier in a mask vector = 0.2) overlaid on the original image, (**o**) the predicted mask (a multiplier in a mask vector = 0.2).

Figure 5 presents examples of segmentation for selected images, including concrete sleepers and *fastener3* elements. Figure 5a–c present segmentation for S multiplier equal to 1. Figure 5d–f show results for S = 0.4, and Figure 5g–i for S = 0.2.

**Figure 6:**
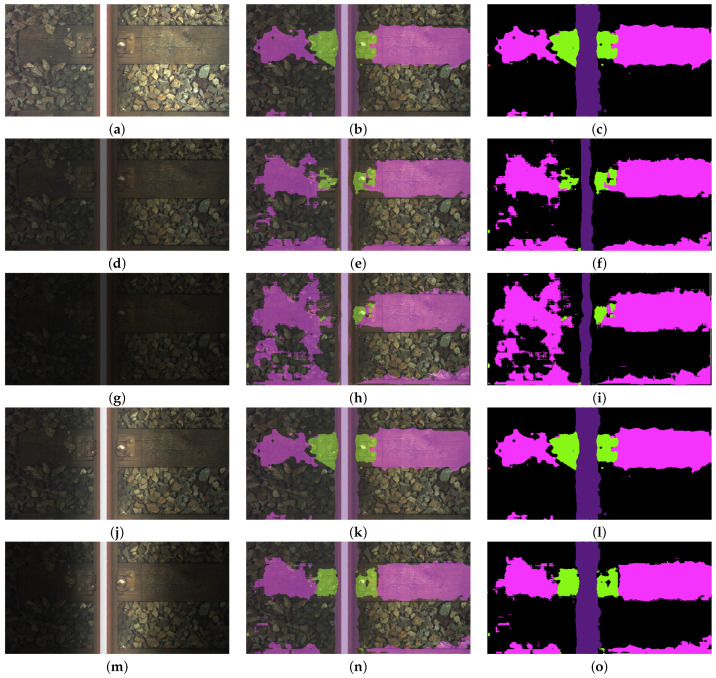
Example results obtained for wooden sleepers and *fastener2* elements: (**a**) the image of the track with S = 1, (**b**) the image with the predicted mask (S = 1) overlaid on the original image, (**c**) the predicted mask (S = 1), (**d**) the image of the track with S = 0.4, (**e**) the image with the predicted mask (S = 0.4) overlaid on the original image, (**f**) the predicted mask (S = 0.4), (**g**) the image of the track with S = 0.2, (**h**) the image with the predicted mask (S = 0.2) overlaid on the original image, (**i**) the predicted mask (S = 0.2), (**j**) the image of the track with the lowest multiplier in a mask vector = 0.4, (**k**) the image with the predicted mask (a multiplier in a mask vector = 0.4) overlaid on the original image, (**l**) the predicted mask (a multiplier in a mask vector = 0.4), (**m**) the image of the track with the lowest multiplier in a mask vector = 0.2, (**n**) the image with the predicted mask (a multiplier in a mask vector = 0.2) overlaid on the original image, (**o**) the predicted mask (a multiplier in a mask vector = 0.2).

Another Figure 6 shows example results obtained for wooden sleepers and *fastener2* elements. Figure 6a–c present segmentation for S multiplier equal to 1. Figure 6d–f show results for S = 0.4, and Figure 6g–i for S = 0.2.

**Figure 7:**
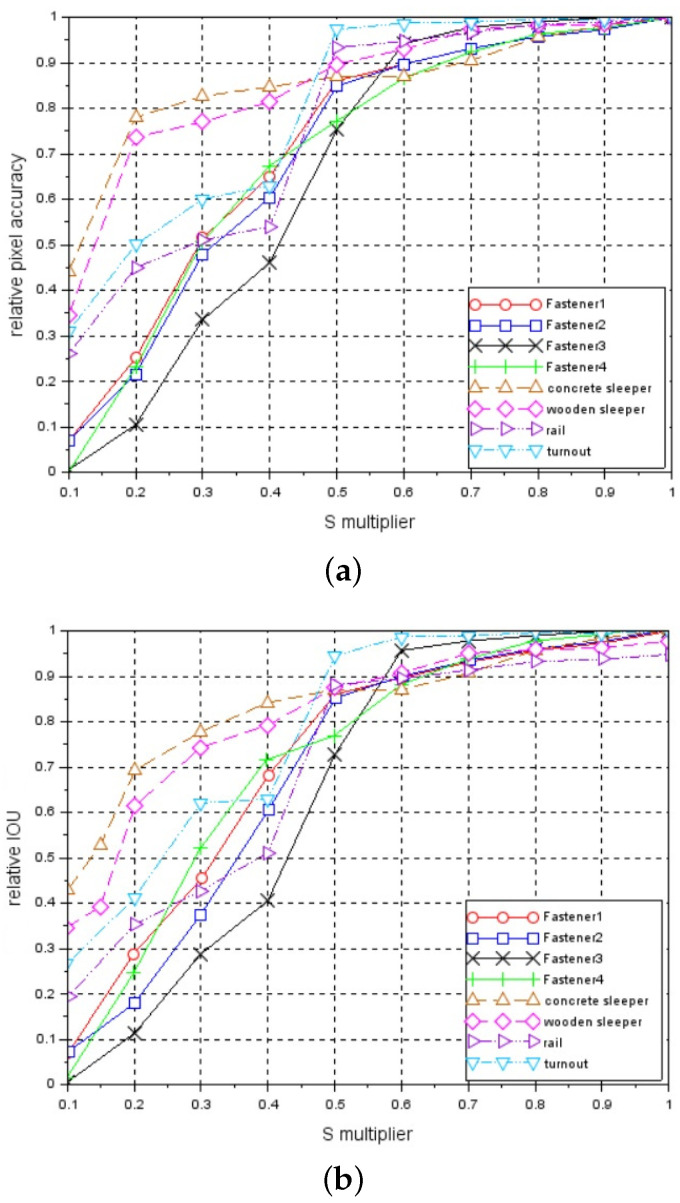
Relationships between relative metrics and S multiplier: (**a**) relative pixel accuracy, (**b**) relative IoU.

Figure 7a presents the relationship between a relative pixel accuracy metric and S multiplier. Figure 7b shows the relationship between a relative IoU metric and S multiplier.

The above two figures show that fasteners are the most sensitive to changes in the brightness level. We also verified the invariance to nonlinear change in the brightness. Because the system is equipped with line-scan cameras and a constant distance between the camera and the light lamp is maintained, we modified the brightness of each single horizontal line of the image according to some constant predefined relationship (mask).

The mask is a vector with a length equal to the width of the image, in which elements are multipliers changing according to Gaussian function. Each row of component V of the HSV model of the processed image is multiplied by the mask vector. In our experiment, we created 8 mask vectors.

**Figure 8:**
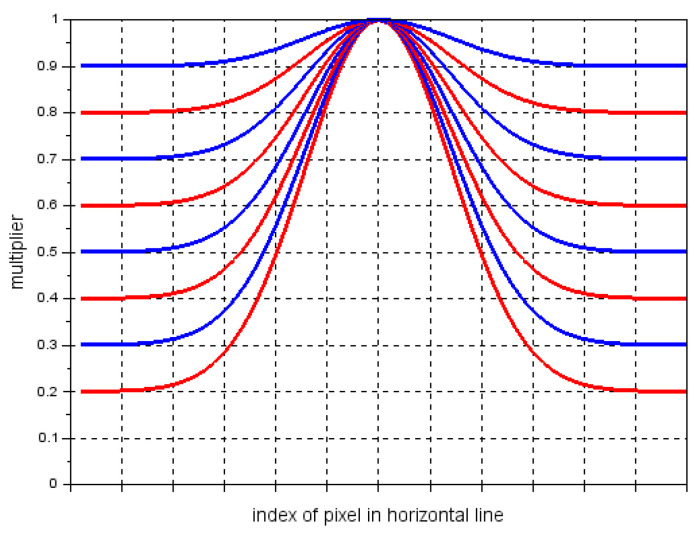
Multiplier distribution for mask vectors.

**Figure 9:**
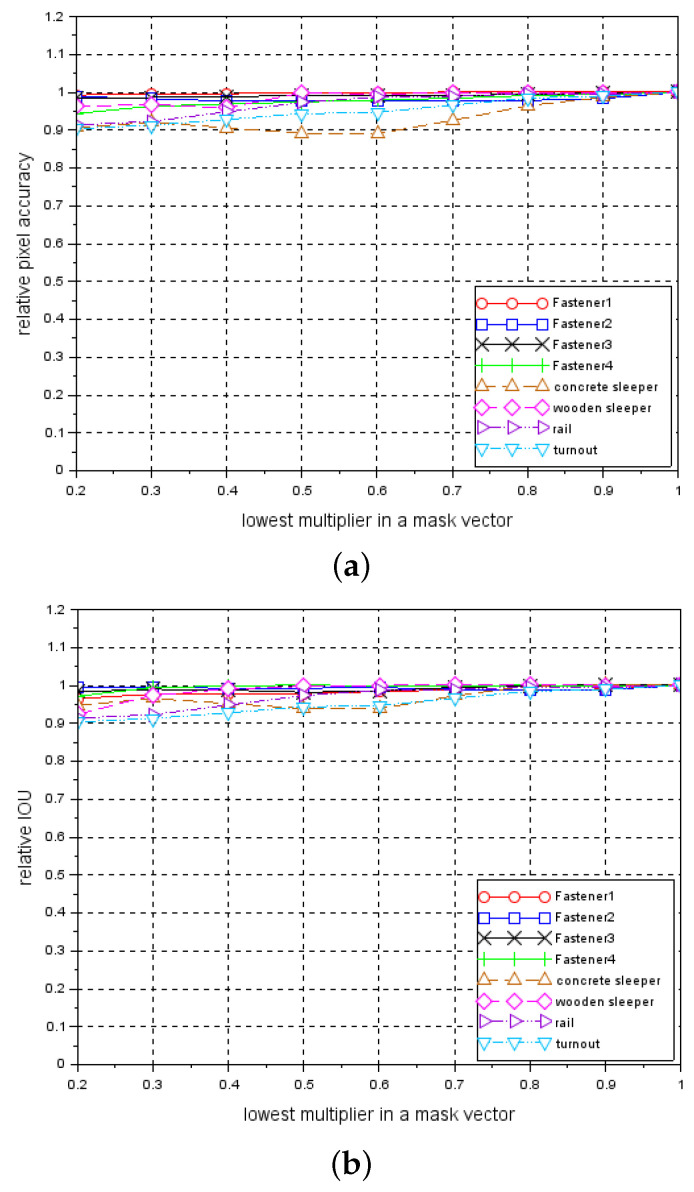
Relationship between relative metrics and mask vectors: (**a**) relative pixel accuracy, (**b**) relative IoU.

These vectors are presented in Figure 8. As in the previous case, for each *test_data* set processed by a mask vector, a relative pixel accuracy and relative IoU are calculated. In addition, in this case, examples of segmentation effects are presented for an image of a track containing a concrete sleeper with *fastener3* elements - Figure 5j–o and an image containing a wooden sleeper with *fastener2* elements; see Figure 6j–o. Figure 9a,b present relative pixel accuracy and relative IoU versus mask vectors, respectively. A single value on horizontal axis corresponds to the lowest multiplier of the mask vector. For example, the value of 0.4 on this axis represents the third mask vector in Figure 8, counting from the bottom. The value of 1.0 corresponds to the unchanged images in the *test_data* set. When we compare Figure 7 with Figure 9, we can see a significant difference in relative pixel accuracy and IoU for fasteners. This is due to the central position of the mask vector relative to the vertical axis of symmetry of the image. Fasteners are also positioned in the vicinity of this vertical axis, which means that the brightness of the fastenings will vary much less than in the case of Figure 7.

## 7. Conclusions

The visual system based on FCN-8 network presented in this paper allows for the detection the following elements of railway track: fasteners, wooden and concrete sleepers, rails, and turnouts. The detection is realized through the image segmentation, where each pixel in the image is assigned the label of the object to which the pixel belongs. This also allows us to determine the degree of the sleeper coverage by the ballast. The image of the track is recorded by two line-scan cameras. Each camera covers half of the area of the track and is connected to separate trained FCN-8 network. Therefore, the track image is processed in parallel by two FCN-8, the first one responsible for the left half of the image and the second one for the right half. Thanks to this, the single-track image is processed at the rate of 15 fps. In Reference [18], the analyzed track image is passed through Fully Convolutional Neural Network and a coarse map of the size of 1/16 input image is a final result. In our approach, FCN-8 network fuses details of information from earlier layers with coarse information from a coarse map, and the result is upsampled 8 times. As a result of this, the predicted mask image is of the size of the input image instead of the size of 1/16 input image as in Reference [18]. We also tested the invariance of our system to changes in brightness of the image. We performed the test both for a constant change in brightness and for a change according to a Gaussian function. The test for a constant change showed that fasteners are moderately sensitive to a change in brightness. A drop in brightness level to 0.7 for fasteners causes a drop in their relative pixel accuracy and IoU to 0.9. The other elements of the railway track are much more insensitive to a change in brightness. In the second test, the brightness is changed according to a Gaussian function in which peak comprises the middle part of the image. Fasteners, rail, turnouts, and, partially, sleepers are also located in the center of the image. This makes the elements of railway track almost insensitive to changes in brightness. A drop in brightness level to 0.2 causes a drop in relative pixel accuracy to 0.9. The system presented here is able to detect the elements of railway track at the rate of 15 fps, which makes it an alternative for other existing systems.

## Figures and Tables

**Table 1:** Pixel accuracy and Processing time for all track elements.

Network	Pixel Accuracy (Formula 3)	Processing Time (ms)
FCN-32	0.69	43
FCN-16	0.78	53
FCN-8	0.891	67

**Table 2:** Pixel accuracy, IoU, Precision, and Recall metrics for track elements.

Track Element	Pixel Accuracy	IoU	Precision	Recall
rail	0.960	0.898	0.91	0.98
wood.sleeper	0.876	0.861	0.89	0.96
concr.sleeper	0.965	0.904	0.905	0.99
fastener1	0.974	0.664	0.84	0.76
fastener2	0.845	0.738	0.87	0.83
fastener3	0.855	0.818	0.86	0.94
fastener4	0.765	0.643	0.77	0.79
turnout	0.923	0.903	0.91	0.99

## Data Availability

Not applicable.
